# Supplementary material for: Quantitative Metabolomic Analysis of Changes in the Rat Blood Serum during Autophagy Modulation: A Focus on Accelerated Senescence
Source: Int J Mol Sci. 2022 Oct 22;23(21):12720. doi: 10.3390/ijms232112720 (PMC9658531; doi:10.3390/ijms232112720)
Supplement: Supplementary file 1 [file ijms-23-12720-s001.zip › ijms-1940018-supplementary.pdf]

Supplementary Information for article

"Quantitative metabolomic analysis of changes in the rat blood serum during autophagy modulation: a focus on accelerated senescence".

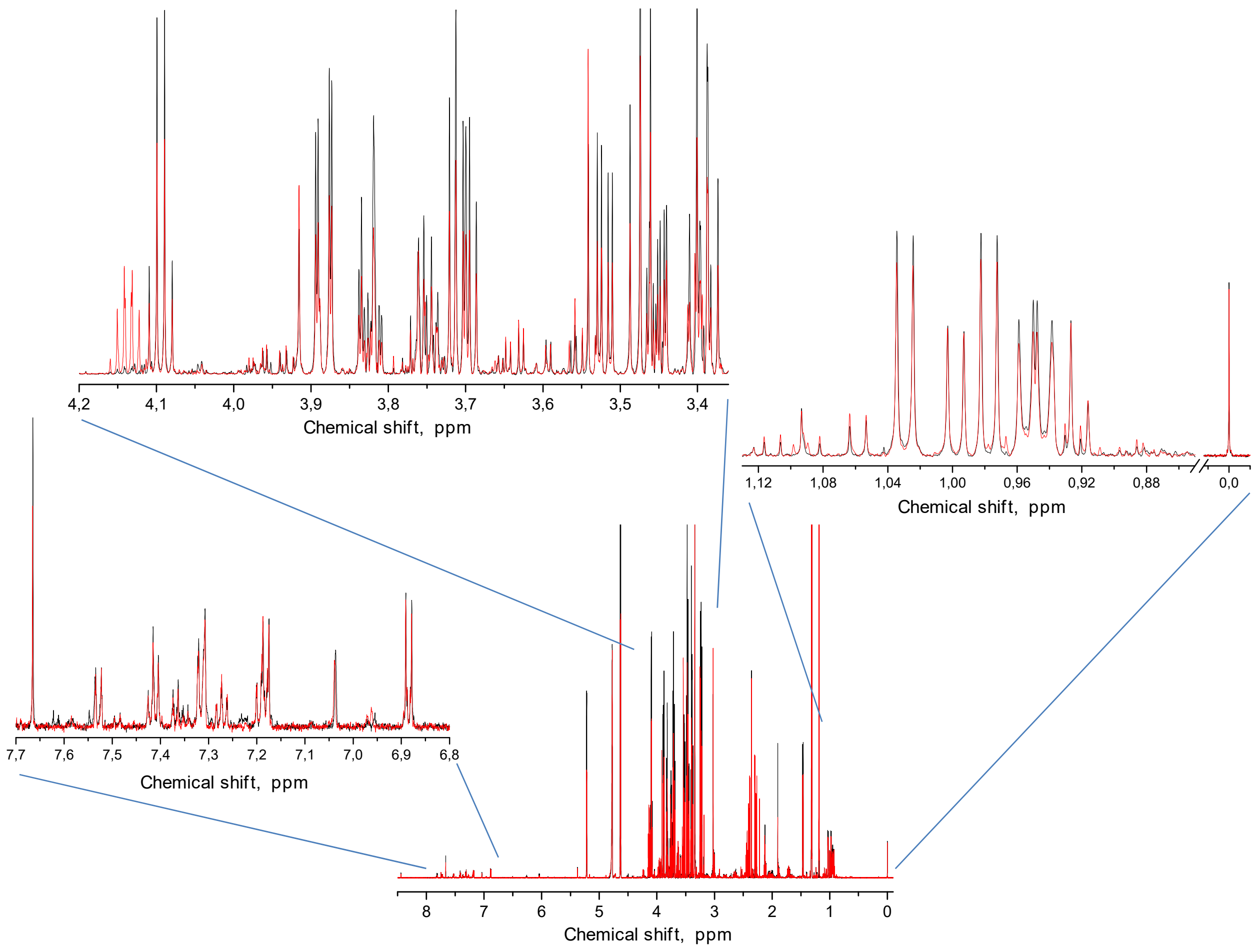

Figure S1. Typical ^{1}H NMR spectrum of a serum extract. The black spectrum is the blood serum sample of the OXYS rat of the basal control group, the red one is the autophagy induction group (fasting for 48 hours).

Table S1. Chemical shifts and multiplicities for metabolites identified and quantified in Wistar, OXYS rats serum by ^{1}H NMR (corresponding to Figure S1).

Metabolite	Chemical shift, ppm	Metabolite	Chemical shift, ppm
2-Hydroxy-3-methyl-butyrate	0.822 (d)	Glutamate	2.342 (m)
2-Hydroxy-butyrate	0.886 (t)	Glutamine	2.127(m), 2.441 (m)
2-Hydroxy-isobutyrate	1.341 (s)	Glycerol	3.546 (dd), 3.64 (dd)
2-Ketoisovalerate	1.112 (d)	Glycine	3.541 (s)
3-hydroxy-butyrate	1.186 (d), 2.289 (dd), 2.4 (dd), 4.136 (m)	Histidine	7.06 (s), 7.8 (s)
3-Methyl-2-oxovalerate	0.881 (t)	Isobutyrate	1.059 (d)
α-Aminobutyrate	0.967 (t)	Isoleucine	0.927 (t), 1.003 (d)
α-Glucose	5.218 (d)	Ketoleucine	0.926 (d)
α-Mannose	5.17 (d)	Lactate	1.318 (d), 4.094 (q)
β-Glucose	4.631 (d)	Leucine	0.954 (dd)
β-Mannose	4.885 (d)	Lysine	3.013 (t)
Acetate	1.902 (s)	Methionine	2.123 (s), 2.62 (t)
Acetoacetate	2.265 (s)	Ornithine	3.042 (t)
Acetone	2.219 (s)	Phenylalanine	7.316 (t), 7.363 (t), 7.416 (t)
Acetyl-carnitine	3.181 (s)	Phosphocholine	3.209 (s)
Alanine	1.466 (d)	Phosphoethanolamine	3.205 (t)
Allantoin	5.372 (s)	Proline	4.118 (dd)
Asparagine	2.841 (dd), 2.941 (dd)	Pyruvate	2.358 (s)
Aspartate	2.803 (dd)	Sarcosine	2.725 (s)
Betaine	3.253 (s)	Serine	3.931 (dd)
Carnitine	3.215 (s)	Succinate	2.391 (s)
Choline	3.189 (s)	Threonine	4.241 (m)
Citrate	2.523 (d), 2.675 (d)	Tryptophan	7.272 (m), 7.307 (s), 7.527 (d), 7.725 (d)
Creatine	3.024 (s), 3.916 (s)	Tyrosine	6.886 (d), 7.181 (d)
Creatinine	3.035 (s)	Uracil	5.788 (d)
Cytidine	6.04 (d), 7.828 (d)	Uridine	5.885 (d), 5.902 (d), 7.86 (d)
Formate	8.442 (s)	Valine	0.978 (d), 1.031(d)
Fumarate	6.504 (s)		

Table S2. Metabolite concentration (μM) in the blood serum of Wistar rats groups employed in autophagy experiments.

Metabolite	Control						Autophagy			
	Basal		PBS injection		Chloroquine injection		Fasting		Chloroquine inhibition	
	Mean ± sd	Min – max	Mean ± sd	Min – max	Mean ± sd	Min – max	Mean ± sd	Min – max	Mean ± sd	Min – max
2-Hydroxy-3-methyl-butyrate	4.3 ± 1.0	3.4 – 6.2	5.0 ± 2.4	3.1 – 8.4	8.1 ± 2.0	5.4 – 11	5.6 ± 1.6	3.9 – 8.2	10 ± 3	6.4 – 13
2-Hydroxy-butyrate	3.2 ± 1.0	2.2 – 4.9	3.0 ± 1.2	1.3 – 4.7	3.6 ± 1.0	2.7 – 4.9	15 ± 5	10 – 23	10.0 ± 1.8	7.9 – 13
2-Hydroxy-isobutyrate	2.9 ± 0.7	1.6 – 3.5	2.8 ± 0.9	2.0 – 4.3	2.6 ± 1.2	1.3 – 4.0	5.1 ± 1.9	2.4 – 7.4	4.9 ± 1.5	3.0 – 6.3
2-Ketoisovalerate	8.1 ± 1.5	6.0 – 10.5	7.3 ± 1.7	5.2 – 9.7	7.6 ± 0.6	6.6 – 8.2	12.3 ± 2.2	9.1 – 15	11.4 ± 1.8	10 – 15
3-hydroxy-butyrate	80 ± 50	46 – 174	90 ± 40	48 – 144	80 ± 40	45 – 142	1900 ± 400	1460 – 2470	720 ± 200	523 – 1031
3-Methyl-2-oxovalerate	6 ± 3	3.1 – 8.7	6.3 ± 1.6	3.8 – 7.9	4.3 ± 0.9	3.1 – 5.5	9 ± 3	4.4 – 12	6.0 ± 1.7	4.4 – 8.9
α-Aminobutyrate	6.5 ± 2.3	2.7 – 9.1	4.4 ± 0.7	3.5 – 5.2	4.7 ± 1.3	3.0 – 6.4	11 ± 3	6.8 – 16	10.3 ± 2.3	5.8 – 12
α-Glucose	2300 ± 600	1617 – 2984	220 ± 210	1864 – 2485	2000 ± 90	1940 – 2156	1200 ± 200	877 – 1344	1700 ± 100	1431 – 1812
α-Mannose	43 ± 12	31 – 63	52 ± 8	41 – 61	70 ± 10	53 – 86	42 ± 5	36 – 48	74 ± 6	66 – 80
β-Glucose	3700 ± 900	2685 – 4906	3700 ± 400	2976 – 4130	3400 ± 200	3281 – 3658	1900 ± 300	1425 – 2223	290 ± 200	2400 – 3008
β-Mannose	21 ± 6	13 – 31	24 ± 5	18 – 29	34 ± 6	26 – 40	19 ± 3	14 – 23	35 ± 3	33 – 41
Acetate	70 ± 20	39 – 93	70 ± 20	46 – 108	60 ± 10	44 – 69	70 ± 14	50 – 90	68 ± 9	54 – 82
Acetoacetate	14 ± 7	7.3 – 26.5	17 ± 13	5.5 – 41	17 ± 7	9.3 – 28	80 ± 90	11 – 243	80 ± 50	31 – 156
Acetone	4 ± 3	1.9 – 10.5	3.6 ± 1.8	1.3 – 5.9	4 ± 3	1.5 – 8.8	30 ± 30	6.2 – 91	26 ± 20	10 – 61
Acetyl-carnitine	22 ± 5	17.3 – 31.7	19.1 ± 2.6	15 – 22	19 ± 4	14 – 24	48 ± 10	36 – 58	44 ± 8	37 – 55
Alanine	470 ± 30	435 – 502	430 ± 60	356 – 507	410 ± 80	312 – 537	330 ± 40	279 – 385	320 ± 40	280 – 364
Allantoin	70 ± 10	56 – 84	60 ± 10	43 – 75	51 ± 5	43 – 58	70 ± 10	49 – 76	54 ± 6	46 – 62
Asparagine	70 ± 10	45 – 85	67 ± 4	60 – 70	50 ± 5	45 – 56	60 ± 10	42 – 72	61 ± 6	53 – 68
Aspartate	47 ± 4	43 – 52	50 ± 20	28 – 76	45 ± 12	30 – 63	49 ± 10	35 – 60	40 ± 20	30 – 71
Betaine	150 ± 10	131 – 162	130 ± 25	100 – 169	60 ± 20	40 – 89	120 ± 30	92 – 158	60 ± 10	52 – 82
Carnitine	50 ± 10	32 – 62	45 ± 7	36 – 56	40 ± 6	36 – 51	27 ± 5	19 – 33	30 ± 5	24 – 35
Choline	22 ± 4	14 – 25	17 ± 3	13 – 23	16 ± 5	12 – 26	15.2 ± 2.2	12 – 17	11.6 ± 1.7	8.8 – 14
Citrate	220 ± 30	190 – 250	160 ± 30	128 – 213	80 ± 20	52 – 99	220 ± 40	152 – 275	90 ± 20	67 – 121
Creatine	280 ± 30	243 – 311	320 ± 80	217 – 432	390 ± 30	335 – 425	400 ± 50	350 – 480	400 ± 60	328 – 482
Creatinine	24 ± 4	19 – 29	22 ± 3	16 – 25	23 ± 3	17 – 26	25 ± 6	14.4 – 31.5	27 ± 3	22 – 31
Cytidine	64 ± 7	56 – 75	67 ± 7	57 – 77	80 ± 4	75 – 86	43 ± 5	35 – 49	39.6 ± 1.9	37 – 42
Formate	28 ± 7	20 – 36	21 ± 4	17 – 27	19 ± 6	12 – 30	22 ± 10	16 – 40	30 ± 20	13 – 69

Fumarate	2.8 ± 1.5	1.6 – 5.8	2.0 ± 1.3	1.2 – 4.7	1.7 ± 0.9	0.79 – 3.1	2.2 ± 0.8	0.95 – 3.11	1.3 ± 0.4	0.75 – 1.8
Glutamate	190 ± 40	141 – 232	130 ± 40	91 – 175	90 ± 20	66 – 121	120 ± 20	88 – 141	90 ± 10	75 – 103
Glutamine	520 ± 70	449 – 607	520 ± 80	418 – 667	490 ± 30	433 – 507	550 ± 30	510 – 611	500 ± 50	433 – 564
Glycerol	110 ± 40	73 – 158	100 ± 50	46 – 159	90 ± 60	35 – 167	160 ± 80	60 – 282	1100 ± 50	70 – 174
Glycine	270 ± 30	234 – 314	290 ± 40	209 – 329	260 ± 30	224 – 290	370 ± 40	298 – 404	310 ± 30	265 – 342
Histidine	56 ± 11	45 – 73	57 ± 7	46 – 65	54 ± 5	49 – 60	48 ± 5	42 – 57	51 ± 7	39 – 59
Isobutyrate	9.8 ± 1.4	8.4 – 12	7.9 ± 1.3	5.7 – 9.6	10.5 ± 1.1	8.6 – 12	17 ± 2	14 – 18	14 ± 3	8.8 – 16
Isoleucine	77 ± 12	67 – 97	72 ± 9	59 – 82	70 ± 10	49 – 83	79 ± 7	71 – 90	88 ± 8	78 – 98
Ketoleucine	3.8 ± 1.5	1.9 – 5.7	3.1 ± 0.6	2.5 – 3.9	3.3 ± 0.6	2.4 – 4.1	7.5 ± 1.8	4.6 – 9.4	5.7 ± 1.6	4.3 – 8.2
Lactate	4800 ± 1200	3059 – 6335	3200 ± 900	2106 – 4294	3100 ± 600	2283 – 4051	3600 ± 600	2821 – 4169	2200 ± 200	2001 – 2527
Leucine	120 ± 20	102 – 158	110 ± 10	95 – 127	120 ± 20	94 – 138	120 ± 10	106 – 141	150 ± 20	122 – 168
Lysine	240 ± 30	201 – 276	230 ± 50	134 – 285	250 ± 20	219 – 272	220 ± 30	193 – 252	250 ± 50	177 – 299
Methionine	50 ± 6	39 – 56	40 ± 6	31 – 44	36 ± 9	27 – 53	33 ± 3	29 – 38	32 ± 8	19 – 43
Ornithine	30 ± 3	26 – 33	40 ± 30	16 – 109	40 ± 20	23 – 69	20 ± 5	13 – 27	27 ± 3	24 – 32
Phenylalanine	66 ± 8	59 – 83	61 ± 8	52 – 77	72 ± 7	66 – 85	61 ± 4	54 – 67	67 ± 9	52 – 76
Phosphocholine	4.3 ± 1.0	3.1 – 5.8	8 ± 7	3.4 – 21	7 ± 10	1.4 – 27	3.9 ± 1.0	2.8 – 5.4	2.4 ± 1.1	1.2 – 4.3
Phosphoethanolamine	70 ± 20	45 – 102	70 ± 40	40 – 136	53 ± 15	35 – 74	52 ± 25	25 – 92	50 ± 20	28 – 89
Proline	150 ± 30	117 – 182	120 ± 10	105 – 139	82 ± 12	69 – 98	44 ± 10	32 – 55	40 ± 8	32 – 52
Pyruvate	220 ± 40	188 – 291	150 ± 40	96 – 200	140 ± 30	92 – 169	250 ± 90	136 – 410	110 ± 30	71 – 140
Sarcosine	1.8 ± 0.2	1.54 – 2.08	2.1 ± 0.4	1.5 – 2.6	1.5 ± 0.3	1.1 – 1.9	1.7 ± 0.4	1.1 – 2.2	1.1 ± 0.2	0.76 – 1.4
Serine	84 ± 9	71 – 95	80 ± 10	64 – 93	79 ± 16	48 – 92	62 ± 6	53 – 69	73 ± 9	64 – 84
Succinate	50 ± 60	23 – 166	27 ± 9	20 – 44	21 ± 8	16 – 36	33 ± 6	24 – 40	19 ± 2	17 – 22
Threonine	210 ± 40	182 – 290	210 ± 30	166 – 249	189 ± 20	169 – 221	180 ± 20	149 – 222	190 ± 20	169 – 230
Tryptophan	90 ± 6	80 – 95	70 ± 2	68 – 74	59 ± 8	49 – 71	77 ± 10	61 – 91	52 ± 7	47 – 64
Tyrosine	70 ± 10	59 – 96	65 ± 7	57 – 73	58 ± 8	49 – 69	60 ± 20	48 – 92	60 ± 10	45 – 73
Uracil	5.7 ± 1.6	3.8 – 7.7	4 ± 3	0.28 – 8.2	4.6 ± 1.8	2.3 – 7.5	5.0 ± 1.4	2.8 – 6.9	6.5 ± 1.2	5.1 – 8.1
Uridine	7.7 ± 1.8	5.0 – 10	9 ± 3	4.7 – 13	7.1 ± 2.3	3.4 – 10	3.6 ± 1.8	1.7 – 6.5	3.9 ± 1.5	2.0 – 5.7
Valine	160 ± 20	141 – 193	140 ± 20	114 – 157	140 ± 20	115 – 172	140 ± 10	126 – 155	170 ± 20	128 – 191

Table S3. Metabolite concentration (μM) in the blood serum of OXYS rats groups employed in autophagy experiments.

Metabolite	Control						Autophagy			
	Basal		PBS injection		Chloroquine injection		Fasting		Chloroquine inhibition	
	Mean ± sd	Min – max	Mean ± sd	Min – max	Mean ± sd	Min – max	Mean ± sd	Min – max	Mean ± sd	Min – max
2-Hydroxy-3-methyl-butyrate	4.9 ± 1.0	4.0 – 6.5	3.9 ± 1.5	2.4 – 6.5	5.7 ± 1.4	3.6 – 7.0	5.2 ± 0.9	4.3 – 6.5	8 ± 3	4.9 – 12
2-Hydroxy-butyrate	3.5 ± 1.3	2.4 – 5.6	4.7 ± 2.3	1.8 – 8.5	4.3 ± 1.3	2.8 – 6.2	14 ± 4	9.1 – 19	15 ± 4	12 – 21
2-Hydroxy-isobutyrate	3.0 ± 0.5	2.6 – 3.9	2.7 ± 0.7	1.6 – 3.8	3.4 ± 1.1	2.4 – 5.1	5.5 ± 1.6	3.6 – 6.8	4.8 ± 0.7	3.6 – 5.6
2-Ketoisovalerate	7.3 ± 1.7	4.4 – 9.3	6.9 ± 1.9	4.6 – 9.1	7.0 ± 0.5	6.3 – 7.8	8.6 ± 2.5	4.6 – 10.3	10.0 ± 2.1	7.5 – 14
3-hydroxy-butyrate	80 ± 20	63 – 114	110 ± 50	77 – 219	130 ± 60	71 – 217	2100 ± 300	1836 – 2373	1500 ± 500	817 – 2065
3-Methyl-2-oxovalerate	5.1 ± 0.8	4.0 – 6.0	5.2 ± 1.6	3.7 – 7.5	3.8 ± 1.2	2.5 – 5.3	6.6 ± 1.5	3.9 – 7.5	5.2 ± 1.9	3.4 – 8.7
α-Aminobutyrate	5.6 ± 2.6	2.6 – 9.5	4.6 ± 1.2	3.1 – 5.8	5.3 ± 1.2	3.9 – 6.9	14 ± 3	12 – 19	14 ± 5	9.4 – 22
α-Glucose	2300 ± 500	1880 – 3188	2600 ± 300	2295 – 3126	2300 ± 200	2090 – 2472	1900 ± 500	1527 – 2770	1700 ± 100	1583 – 1858
α-Mannose	40 ± 8	29 – 53	39± 3	35 – 43	60 ± 10	48 – 74	50 ± 6	46 – 60	57 ± 9	45 – 65
β-Glucose	3800 ± 900	3056 – 5277	430 ± 600	3841 – 5196	3800 ± 300	3490 – 4150	3100 ± 900	2502 – 4616	2900 ± 200	2665 – 3139
β-Mannose	19 ± 4	13 – 23	21 ± 2	18 – 23	28 ± 4	24 – 33	26 ± 3	22 – 30	26 ± 5	19 – 32
Acetate	80 ± 40	58 – 152	100 ± 30	66 – 146	70 ± 20	57 – 99	90 ± 20	68 – 107	70 ± 10	56 – 95
Acetoacetate	13 ± 4	9.1 – 19	11 ± 6	6.2 – 22	17 ± 8	7.6 – 25	100 ± 70	20 – 162	120 ± 110	18 – 290
Acetone	3.9 ± 1.3	2.1 – 5.4	3.8 ± 2.5	2.2 – 8.6	6 ± 6	1.3 – 17	30 ± 10	20 – 47	27 ± 9	16 – 38
Acetyl-carnitine	21± 3	17 – 25	24 ± 4	18 – 29	23 ± 4	18 – 28	60 ± 8	51 – 70	50 ± 10	38 – 65
Alanine	440 ± 50	366 – 508	430 ± 40	389 – 495	380 ± 40	341 – 428	370 ± 20	340 – 384	340 ± 30	268 – 361
Allantoin	62 ± 5	55 – 69	58 ± 4	53 – 64	60 ± 10	46 – 81	64 ± 8	56 – 75	59 ± 9	47 – 72
Asparagine	66 ± 9	57 – 77	60 ± 10	44 – 78	55 ± 6	47 – 60	64 ± 10	54 – 79	54 ± 6	46 – 63
Aspartate	50 ± 10	40 – 68	51 ± 15	30 – 68	50 ± 10	37 – 68	50 ± 20	35 – 74	50 ± 20	31 – 67
Betaine	130 ± 30	103 – 169	170 ± 30	133 – 220	80 ± 20	63 – 108	140 ± 30	115 – 192	80 ± 30	57 – 127
Carnitine	46 ± 8	35 – 55	45 ± 6	36 – 52	47 ± 6	41 – 54	32 ± 6	27 – 43	33 ± 5	28 – 40
Choline	19.0 ± 0.5	18.1 – 19.6	19 ± 3	16 – 24	15 ± 4	11 – 22	15 ± 4	10 – 20	12 ± 3	9.6 – 16
Citrate	160 ± 20	132 – 174	140 ± 20	110 – 176	80 ± 20	56 – 104	180 ± 20	159 – 192	110 ± 20	78 – 133
Creatine	310 ± 40	257 – 380	350 ± 70	295 – 496	410 ± 50	347 – 452	450 ± 60	390 – 544	440 ± 30	392 – 487
Creatinine	23 ± 2	20.1 – 25.8	24 ± 3	20 – 28	29 ± 5	23 – 35	28 ± 2	26 – 32	29 ± 4	23 – 33
Cytidine	54 ± 1	53 – 55	54 ± 3	48 – 58	78 ± 5	73 – 86	30 ± 2	28 – 33	31 ± 6	26 – 39
Formate	26 ± 9	18 – 41	22 ± 6	13 – 28	20 ± 10	11 – 51	30 ± 10	15 – 49	30 ± 10	12 – 46

Fumarate	1.7 ± 0.7	1.1 – 2.7	2.2 ± 1.3	1.2 – 4.8	1.1 ± 0.5	0.06 – 1.6	2.2 ± 0.4	1.8 – 2.8	1.5 ± 0.4	1.1 – 2.0
Glutamate	140 ± 10	118 – 157	150 ± 10	127 – 161	100 ± 10	85 – 125	140 ± 20	119 – 165	100 ± 10	80 – 113
Glutamine	500 ± 50	431 – 541	510 ± 50	422 – 556	470 ± 40	426 – 517	570 ± 40	531 – 633	480 ± 40	433 – 556
Glycerol	110 ± 60	60 – 211	110 ± 70	61 – 211	100 ± 60	61 – 174	140 ± 60	70 – 211	150 ± 90	75 – 302
Glycine	290 ± 20	262 – 314	290 ± 20	269 – 332	280 ± 30	248 – 320	440 ± 30	402 – 492	360 ± 40	305 – 417
Histidine	62 ± 4	57 – 66	61 ± 7	50 – 71	56 ± 5	49 – 64	58 ± 1	56 – 60	56 ± 4	49 – 60
Isobutyrate	9.3 ± 0.7	8.1 – 10.2	9.6 ± 1.9	7.8 – 13	8.9 ± 0.8	7.8 – 10.2	15 ± 3	12 – 18	14 ± 2	11 – 16
Isoleucine	82 ± 8	75 – 98	80 ± 10	68 – 104	80 ± 10	71 – 95	97 ± 6	91 – 105	110 ± 10	89 – 123
Ketoleucine	3.0 ± 0.8	1.6 – 3.8	3.1 ± 1.0	1.5 – 4.5	2.3 ± 0.6	1.3 – 3.2	5.4 ± 1.5	3.2 – 7.4	4.7 ± 1.8	3.1 – 7.5
Lactate	3400 ± 600	2257 – 3977	3700 ± 600	2582 – 4180	3100 ± 700	2116 – 3920	2800 ± 700	1740 – 3409	2300 ± 400	1743 – 2943
Leucine	140 ± 20	111 – 176	140 ± 20	112 – 174	130 ± 20	108 – 151	144 ± 5	140 – 152	160 ± 20	138 – 191
Lysine	230 ± 30	203 – 271	220 ± 20	192 – 261	240 ± 40	203 – 316	270 ± 30	241 – 323	250 ± 20	206 – 276
Methionine	47 ± 3	44 – 52	45 ± 7	35 – 53	36 ± 4	30 – 39	35 ± 2	32 – 38	33 ± 4	27 – 37
Ornithine	26 ± 6	22 – 38	32 ± 7	23 – 40	27 ± 5	21 – 36	26 ± 4	22 – 32	28 ± 4	23 – 34
Phenylalanine	65 ± 7	55 – 74	64 ± 9	52 – 79	71 ± 8	60 – 81	65 ± 2	63 – 69	68 ± 6	61 – 75
Phosphocholine	3.3 ± 0.5	2.8 – 4.1	3.4 ± 1.7	1.7 – 6.2	2.3 ± 0.6	1.6 – 3.0	4.6 ± 2.1	2.6 – 7.9	2.5 ± 0.8	1.3 – 3.6
Phosphoethanolamine	80 ± 20	60 – 95	70 ± 20	53 – 108	60 ± 20	24 – 81	70 ± 20	46 – 95	60 ± 20	39 – 83
Proline	130 ± 10	123 – 149	130 ± 30	84 – 160	80 ± 20	47 – 100	50 ± 5	46 – 58	36 ± 5	31 – 42
Pyruvate	180 ± 30	149 – 233	120 ± 30	93 – 179	130 ± 30	101 – 176	200 ± 40	125 – 235	110 ± 30	70 – 142
Sarcosine	2.8 ± 0.5	2.3 – 3.5	3.1 ± 0.8	1.7 – 3.7	2.1 ± 0.8	1.3 – 3.3	2.0 ± 0.2	1.7 – 2.3	1.3 ± 0.4	0.7 – 1.8
Serine	80 ± 7	68 – 86	100 ± 10	90 – 115	70 ± 20	50 – 105	60 ± 10	49 – 75	78 ± 8	64 – 85
Succinate	26 ± 7	17 – 35	50 ± 40	22 – 123	20 ± 3	16 – 24	35 ± 10	28 – 52	24 ± 4	20 – 30
Threonine	200 ± 20	182 – 220	220 ± 30	188 – 279	190 ± 20	161 – 236	230 ± 10	210 – 241	210 ± 20	190 – 240
Tryptophan	79 ± 9	64 – 89	90 ± 10	74 – 99	61 ± 8	50 – 75	80 ± 8	72 – 94	57 ± 3	53 – 61
Tyrosine	78 ± 5	72 – 85	79 ± 9	67 – 94	70 ± 10	58 – 87	70 ± 10	62 – 84	70 ± 10	55 – 84
Uracil	4.3 ± 2.1	2.1 – 7.2	3.7 ± 2.3	0.20 – 6.52	5.0 ± 2.0	3.4 – 8.5	4.5 ± 1.3	2.7 – 5.9	5.4 ± 2.1	2.2 – 7.9
Uridine	7.0 ± 1.0	5.9 – 8.2	7.9 ± 1.2	6.0 – 9.5	8 ± 3	4.7 – 13	3.8 ± 2.2	1.5 – 6.8	2.5 ± 1.3	0.7 – 4.2
Valine	170 ± 20	150 – 198	160 ± 30	135 – 210	150 ± 20	129 – 188	173 ± 6	163 – 179	180 ± 20	156 – 203

Table S4. Groups of animals employed in autophagy experiments.

Number of animals	Control			Autophagy	
	Intact	Injection		Fasting	Chloroquine inhibition
		PBS	Chloroquine		
Wistar	6	6	6	6	6
OXYS	6	6	6	5	6

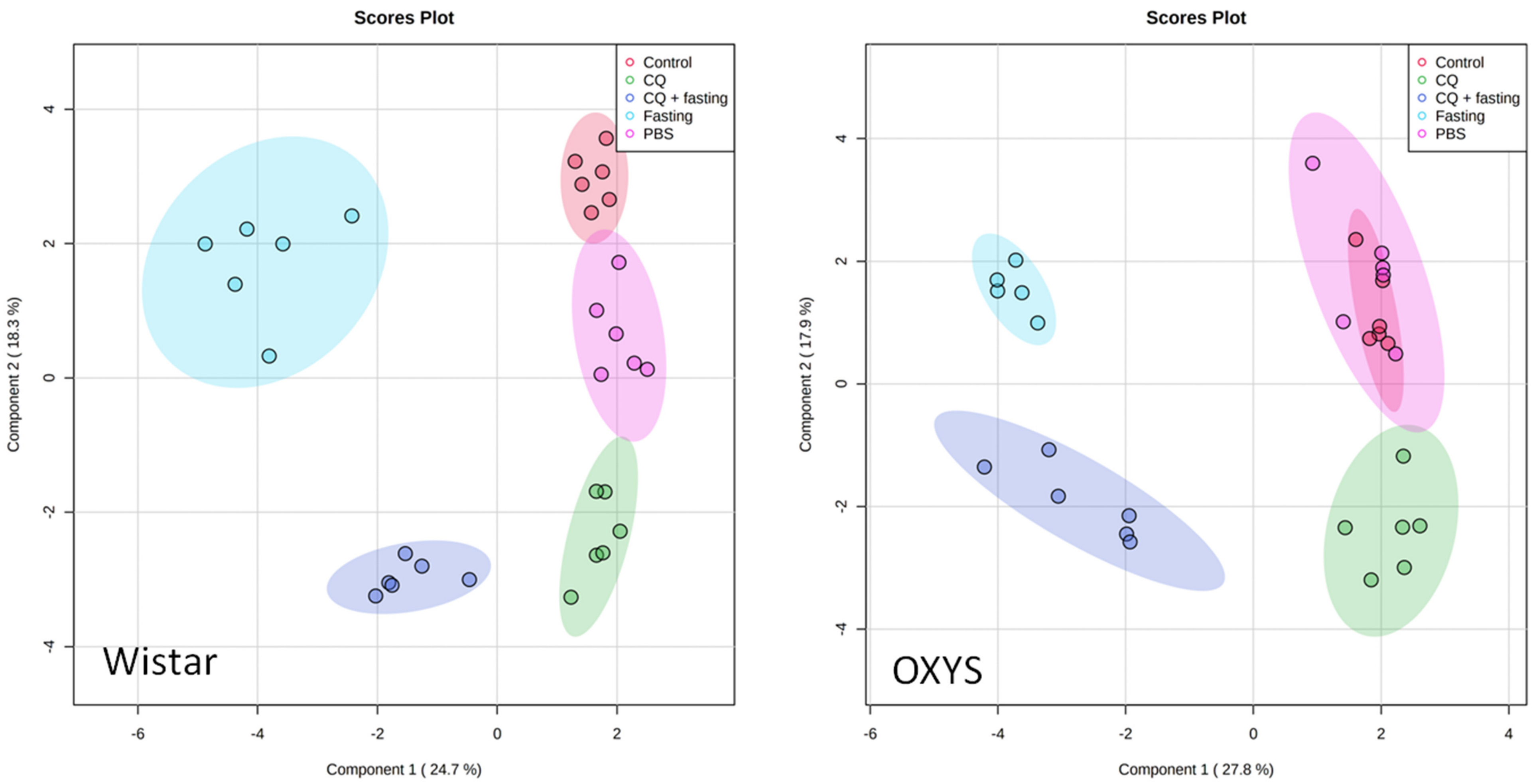

Figure S2. sPLS-DA score plot for concentration of aqueous metabolites extracted from blood serum of two rats strain. The groups are presented according to experimental design (Figure 1, Table S4).

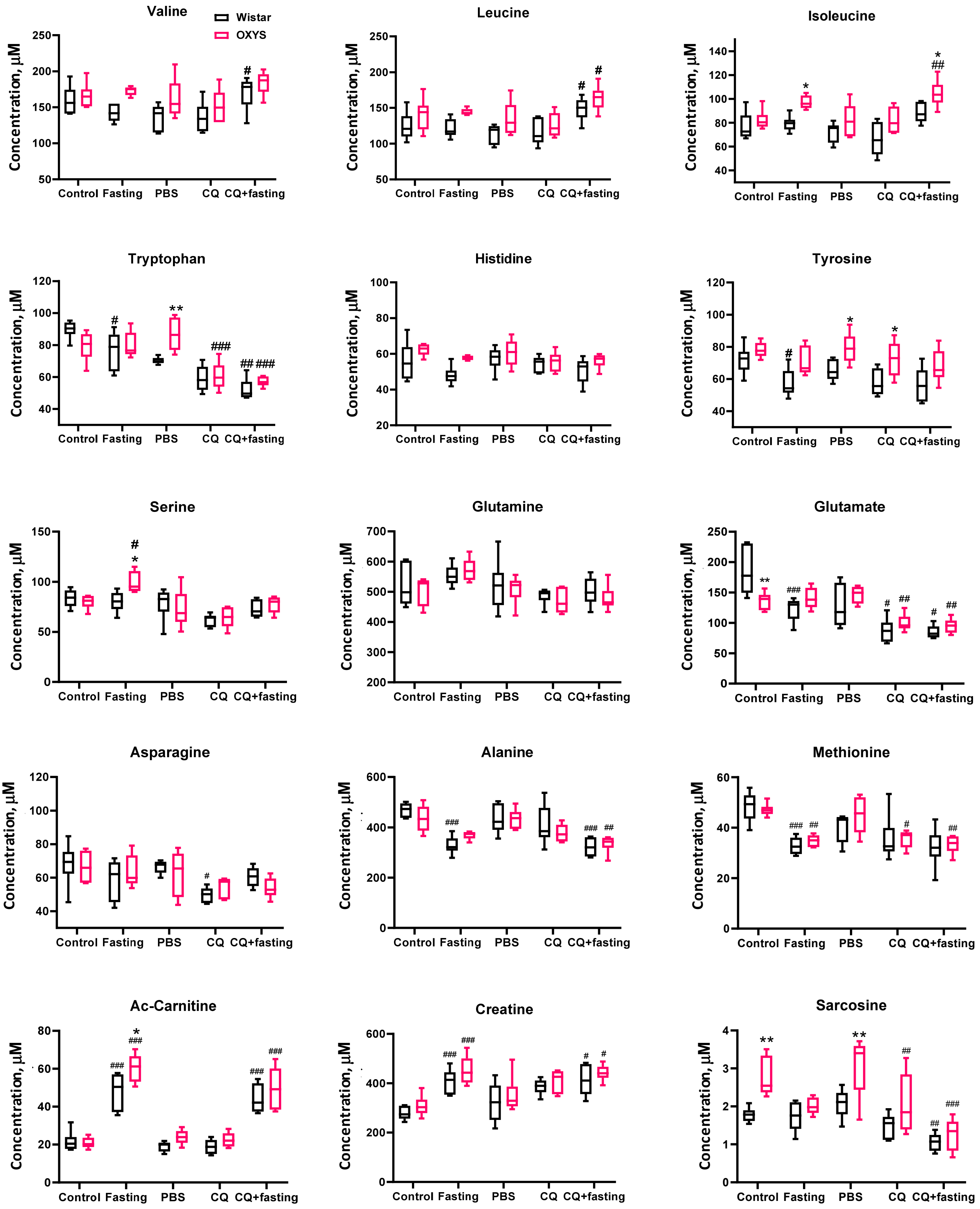
Valine
Wistar
OXYS
Leucine
Isoleucine
Tryptophan
Histidine
Tyrosine
Serine
Glutamine
Glutamate
Asparagine
Alanine
Methionine
Ac-Carnitine
Creatine
Sarcosine
Concentration, µM
Control Fasting PBS CQ CQ+fasting

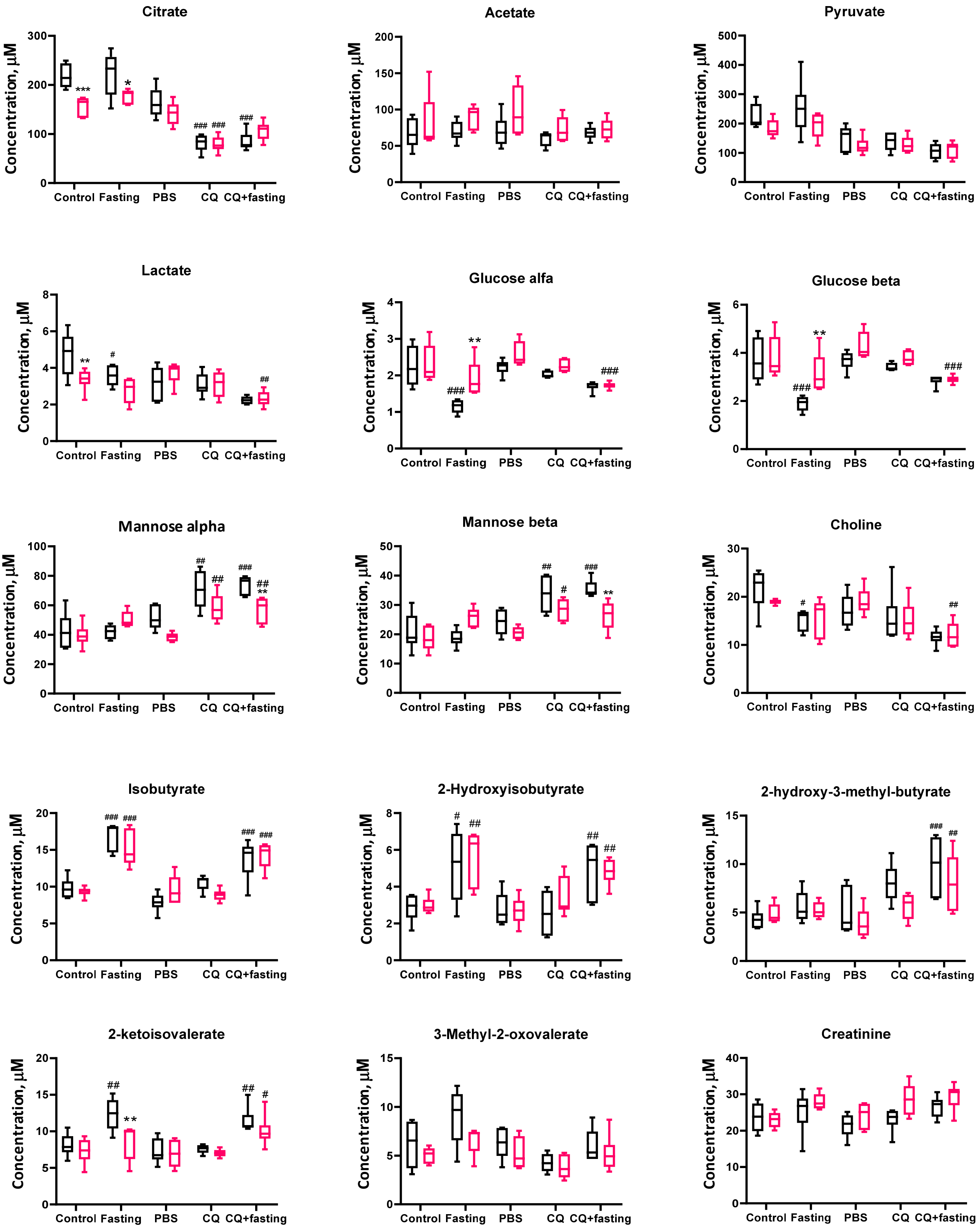

Figure S3. Concentration of serum metabolites for Wistar (black boxes) and OXYS (pink boxes) rats under autophagy modulation by 48 hour fasting, chloroquine (CQ) treatment and combined effects of fasting and CQ Data presented by boxplots: medians, the 25–75% interquartile range (bars), and min–max (error bars). Intact control serves as a control group in the case of fasting and phosphate-buffered saline (PBS) group serves as a vehicle control to CQ and CQ+fasting treatment groups. Notation means: * $p < 0.05$, ** $p < 0.01$, *** $p < 0.001$ OXYS *vs* Wistar rats from the same treatment group ; # $p < 0.05$, ## $p < 0.01$, ### $p < 0.001$ *vs* control group (intact control in the case of fasting, PBS vehicle control in the case of CQ/CQ+fasting) within the same strain of rats, post-hoc comparisons after ANOVA or Kruskall–Wallis test.

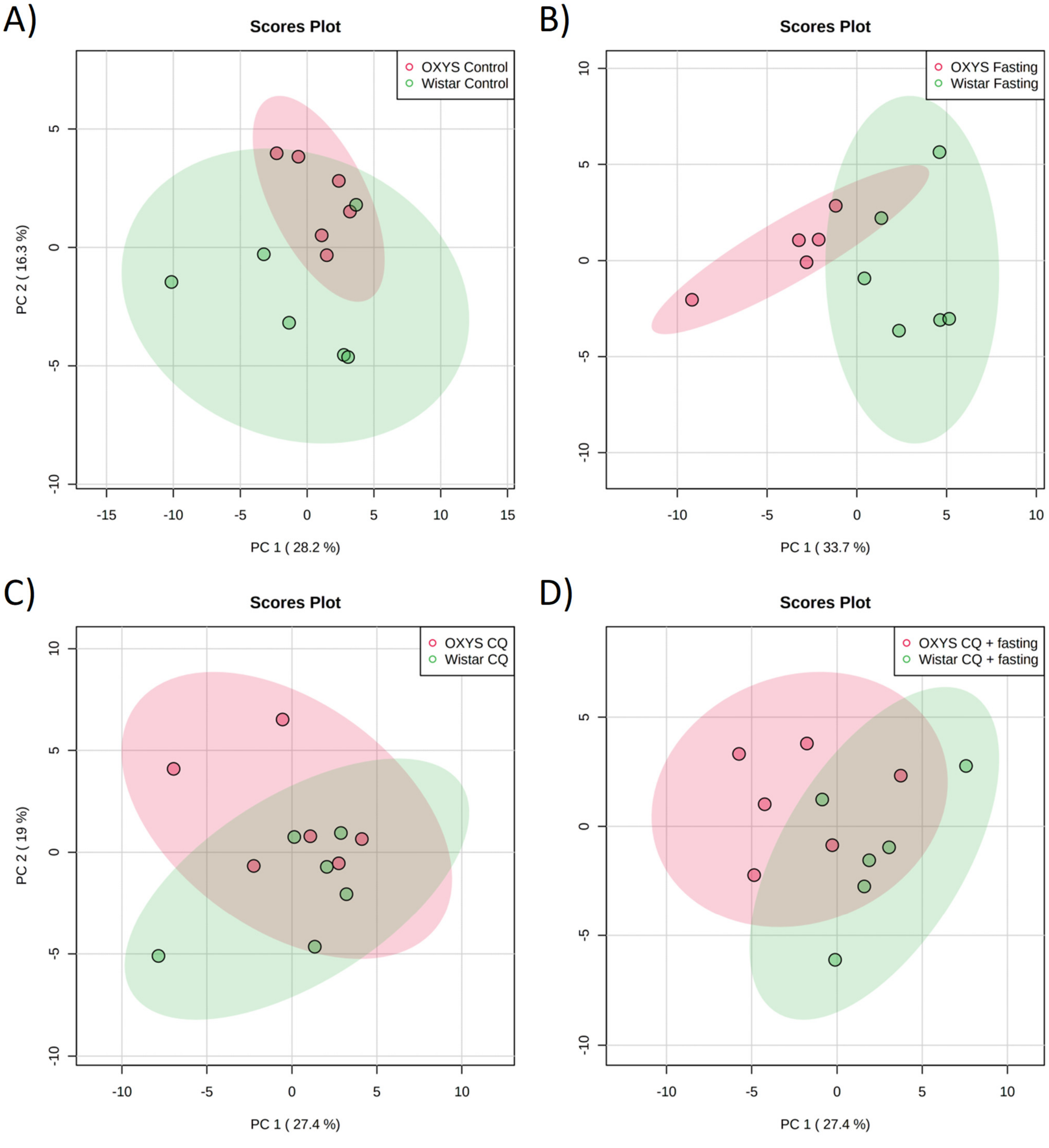

Figure S4. Changes of metabolomic profile depending on the genotype. PCA scores plots of serum metabolomic profiles of Wistar and OXYS rat strains for experimental groups: control (A), fasting (B), CQ treated (C), fasting + CQ treated (D).
